# Monoaxial Pedicle Screws Are Superior to Polyaxial Pedicle Screws and the Two Pin External Fixator for Subcutaneous Anterior Pelvic Fixation in a Biomechanical Analysis

**DOI:** 10.1155/2013/683120

**Published:** 2013-12-03

**Authors:** Rahul Vaidya, Ndidi Onwudiwe, Matthew Roth, Anil Sethi

**Affiliations:** Detroit Medical Center, Orthopedics Department, Wayne State School of Medicine, 4D UHC, St. Antoine Street, 540 E Canfield Street, Detroit, MI 48201, USA

## Abstract

*Purpose*. Comparison of monoaxial and polyaxial screws with the use of subcutaneous anterior pelvic fixation. *Methods*. Four different groups each having 5 constructs were tested in distraction within the elastic range. Once that was completed, 3 components were tested in torsion within the elastic range, 2 to torsional failure and 3 in distraction until failure. *Results*. The pedicle screw systems showed higher stiffness (4.008 ± 0.113 Nmm monoaxial, 3.638 ± 0.108 Nmm Click-x; 3.634 ± 0.147 Nmm Pangea) than the exfix system (2.882 ± 0.054 Nmm) in distraction. In failure testing, monoaxial pedicle screw system was stronger (360 N) than exfixes (160 N) and polyaxial devices which failed if distracted greater than 4 cm (157 N Click-x or 138 N Pangea). The exfix had higher peak torque and torsional stiffness than all pedicle systems. In torsion, the yield strengths were the same for all constructs. *Conclusion*. The infix device constructed with polyaxial or monoaxial pedicle screws is stiffer than the 2 pin external fixator in distraction testing. In extreme cases, the use of reinforcement or monoaxial systems which do not fail even at 360 N is a better option. In torsional testing, the 2 pin external fixator is stiffer than the pedicle screw systems.

## 1. Introduction

A technique of subcutaneous anterior pelvic fixation for anterior pelvic ring fractures has been recently reported and has been termed “infix” [1]. It involves two supra-acetabular pins [2–5] and a subcutaneous rod, tunneled under the skin, at the top of the “bikini area” [6, 7]. In a multicenter study, infix has been shown to be effective in the treatment of pelvic fractures when combined with the appropriate posterior fixation. It has good patient tolerance, avoids the traditional complications of external fixation [8], and is useful to reduce pelvic injuries [9, 10]. When constructed with traditional polyaxial pedicle screws, infix is biomechanically as effective at posterior SI compression as a femoral distracter [11] and stiffer than a traditional 2 pin anterior external fixator in single stance gait testing in synthetic pelvic models [11, 12].

**Figure 1:**
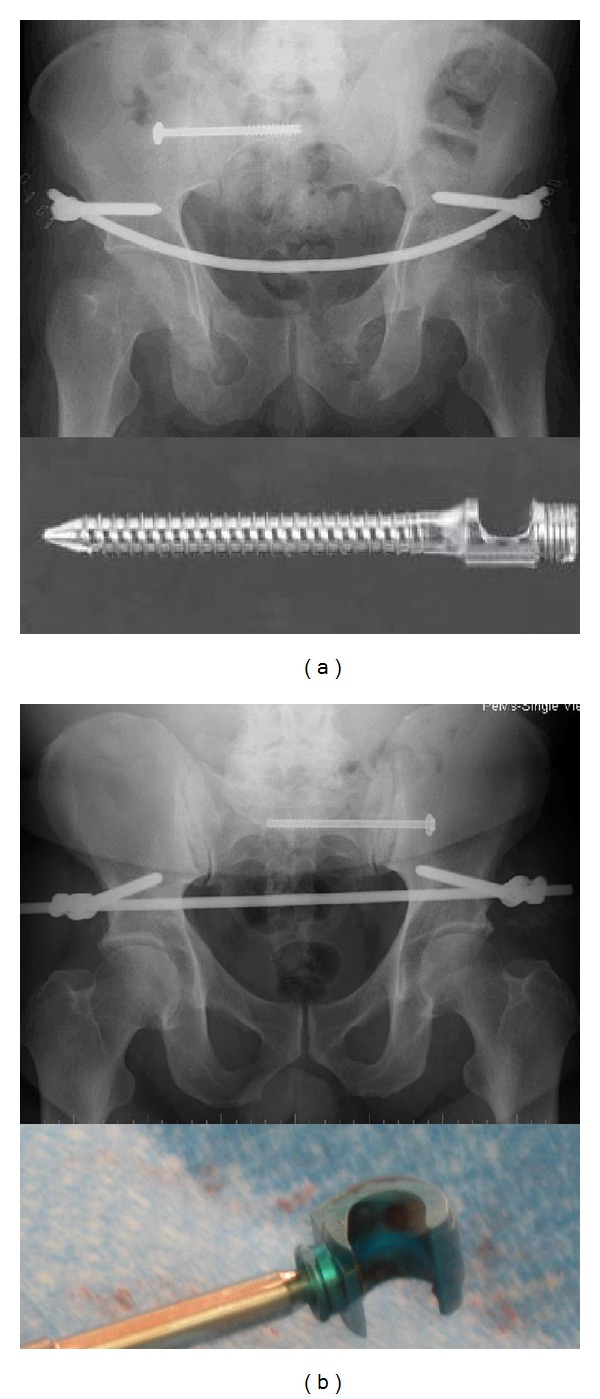
X-rays with an (infix) with (a) a monoaxial system and (b) a polyaxial system reinforced with C-rings.

Pedicle screw implants use a rod screw construct which can be made with monoaxial screws where the head of the pedicle screw is immobile or polyaxial screws where the head of the pedicle screw can rotate in several directions. Polyaxial screws allow surgeons maneuverability when applying these devices, so that the screw heads do not have to line up exactly to attach the rod. The maneuverability of these screws is at the expense of some strengths of the construct. We have used monoaxial screws to construct the infix device in extremely unstable situations such as APC3 injuries or used C-rings (Synthes Spine, USA) to reinforce polyaxial constructs with good success (Figure 1). However there have been reports of failures of the infix construct in 3 APC injuries in a the multicenter series requiring revision surgery [8] and in another case report [11] all using polyaxial screws.

The purpose of this study is to evaluate the stiffness, and strength to failure of a supra-acetabular internal fixation construct when constructed with either monoaxial or polyaxial screws and compare it with a standard two pin external fixation device. The constructs were tested in in polyethelene blocks, as it was found that testing to failure in synthetic pelvic models or cadavers failed at the screw bone interface making it difficult to test the devices to extremes. This is the standard material that is used for pedicle screw construct testing in cyclic loading and failure for the US Food and Drug Administration.

## 2. Methods

**Figure 2:**
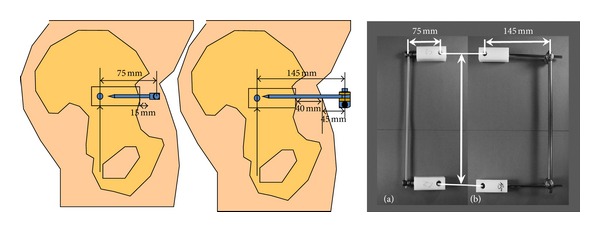
Measurements of the set-up for testing in polyethylene blocks.

**Figure 3:**
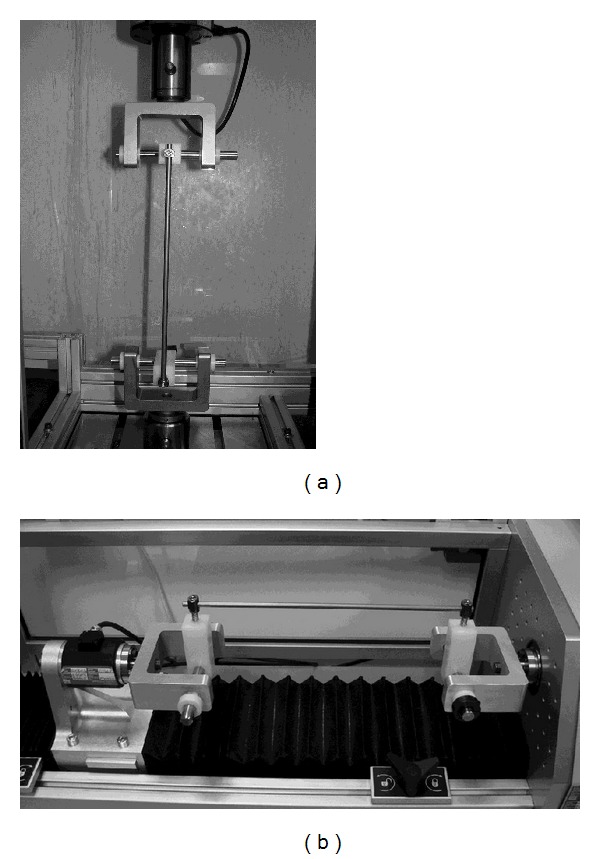
Set-up for testing. (a) Distraction MTS RT/50 Electromechanical Test. (b) Torsion MTS Bionix Electromechanical Torsion Test Frame (MTS Corp., Eden Prairie, MN).

Six millimeter Schanz screws, an 11 mm carbon fiber rod, and external fixation clamps were used for the two pin external fixator system (Synthes, USA). The pedicle screw systems employed 7 mm × 55 mm titanium screws and a 6 mm titanium rod (Synthes Spine, USA). These are the commercially available standard implants that can be used for each device. All screws were inserted into ultra-high molecular weight polyethylene (UHMWPE) blocks measuring 75 mm × 25 mm × 25 mm. (Figure 2). The dimensions were created to mimic the normal pelvis from CT evaluations of both devices in human subjects [6] and previous biomechanical studies in a synthetic bone models [10, 11]. The constructs were assembled to have an active longitudinal length of 280 mm. For the pedicle screw systems, a gap of 15 mm was left between the screw head and test block, resulting in a construct moment arm of 75 mm. This is the distance that these screws would have to sit above the bone in a human case (Figure 3). The external fixator (exfix) constructs were assembled with a 145 mm construct moment arm. This included 60 mm in the block, 40 mm of soft tissue from the pelvic bone to the skin, and another 45 mm above the skin where the clamps would attach.

### 2.1. Testing Procedures

Four different constructs were tested in this study with each group having five constructs for a total of twenty that were tested. These included (1) Large External Fixation System (Synthes, West Chester, PA), (2) monoaxial pedicle screw system (Universal Spinal System, Synthes, West Chester, PA), (3) Polyaxial Pedicle Screw System 1 (Click-x Pedicle Screw System, Synthes, West Chester, PA), (4) Polyaxial Pedicle Screw System 2 (Pangea Pedicle Screw System, Synthes, West Chester, PA).

**Figure 4:**
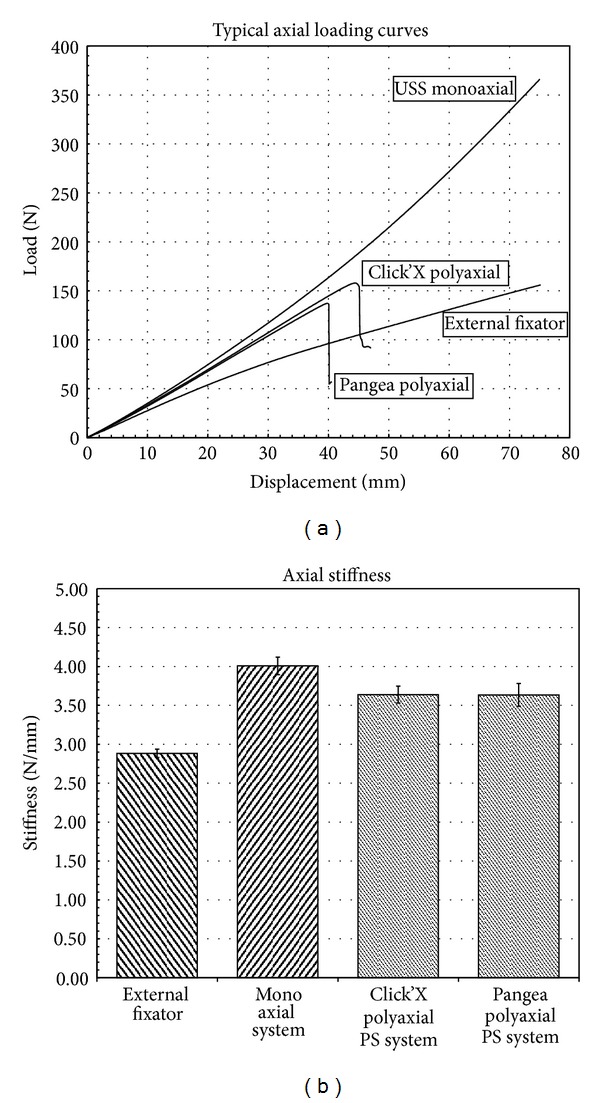
(a) Load displacement curves for axial testing which show that the polyaxial systems failed after 40 mm of displacement. (b) The polyaxial screws were slightly less stiff than the monoaxial system and all three-screw systems were stiffer than the external fixator.

All four constructs with 5 samples each were tested first in distraction in the elastic range (20 mm of displacement). Once distraction testing was completed, all components were tested in torsion (Figure 4) within the elastic range (10°). Two components from each construct group were retested to torsional failure (or 60°). The remaining three components in each construct group were then retested in distraction until failure (or 75 mm).

Distraction was performed on an MTS RT/50 Electromechanical Test Frame (MTS Corp., Eden Prairie, MN). Standard clevis fixtures were rigidly attached to the load cell and lower platen of the test machine. Constructs were mounted to the clevis fixtures using 12.7 mm diameter steel hinge pins. An axial distraction load was applied to the construct at a test speed of 5 mm/min. Load-displacement curves were acquired for each construct and bending yield load, stiffness, and ultimate bending failure load were calculated, as applicable. Yield load calculations were based on 0.020 times the active length.

Torsional testing was performed on an MTS Bionix Electromechanical Torsion Test Frame (MTS Corp., Eden Prairie, MN). Clevis fixtures that prevented rotation of the test block were rigidly attached to the load cell and lower platen of the test machine. Constructs were mounted to the clevis fixtures using 12.7 mm diameter steel hinge pins. Spacers, that prevent test block rotations about the hinge pins, were manually set. Angular displacement was applied to the construct at a test speed of 60° per min, up to a maximum of 60°. Axial load was maintained at 0 N. Torque-angular displacement curves were acquired for each construct tested and torsional yield load, stiffness, and ultimate torque were calculated, if applicable. Yield torque was based on a 5° offset.

### 2.2. Statistics

#### 2.2.1. Methodology

In this experiment, comparisons of different treatments to a single control (exfix) were desired. Thus, there are only *a* − 1 comparisons to be made where *a* − 1 is the number of treatments (excluding the control). The appropriate statistical analysis in this case which controls the overall type I error rate, *a*, is Dunnett's Test [13, 14]. The hypotheses of interest are

(1) $$\begin{array}{c} H_{0}\text{ : }\mu_{i}=\mu_{a},\\ H_{1}\text{ : }\mu_{i}\neq \mu_{a} \end{array}$$

for *i* = 1,2, 3,…, *a* − 1. Dunnett's procedure is a modification of the usual *t*-test. For each hypothesis, the observed differences were computed in the sample means and they reject the null hypothesis if

(2) $$\begin{array}{c} \left|{{\overset{-}{y}}_{i}}_{\cdot }-{\overset{-}{y}}_{a\cdot }\right|>d_{\alpha }\left(a-1,f\right)\sqrt{\text{MSE}\left(\frac{1}{n_{i}}+\frac{1}{n_{a}}\right)}, \end{array}$$

where MSE is the mean square error from a one-way ANOVA and the constant, *d*_*α*_(*a* − 1, *f*), requires special tables and takes into account the degrees of freedom (*f*), number of treatments (*a* − 1), and the type I error rate, *α*. The data was analyzed using Dunnett's Test using a type I error of *α* = 0.05. Using (2), if the absolute value of any paired difference of means between a treatment and control exceeds 0.071 for torsion data or 0.181 for axial data, then the difference is significant.

## 3. Results

### 3.1. Distraction Testing in the Elastic Range

The monoaxial construct was significantly stiffer (4.008 ± 0.113 Nmm) in distraction in the elastic range than the polyaxial systems (Click-x 3.638 ± 0.108 Nmm; Pangea Poly 3.634 ± 0.147 Nmm) and the external fixator (2.882 ± 0.054 Nmm) (*P* < .05). Both polyaxial constructs were significantly stiffer in distraction in the elastic range than the external fixator (*P* < .05) (Figure 4).

### 3.2. Distraction Testing to Failure

No failures were observed in either the external fixator or monoaxial pedicle screw group when tested up to 75 mm of displacement. Therefore, when tested to failure, the peak loads reported correspond to the maximum load at that displacement which was 360 N for the monoaxial construct and 160 N for the external fixator (Figure 4). The polyaxial (Click-x and Pangea) systems both failed at 40–50 mm displacement. This was due to the rod slipping in the screw head. Yield loads were 157 ± 4 N at 45 mm for Click-x and 138 ± 6 N at 40 mm for Pangea poly.

### 3.3. Torsion Testing

**Figure 5:**
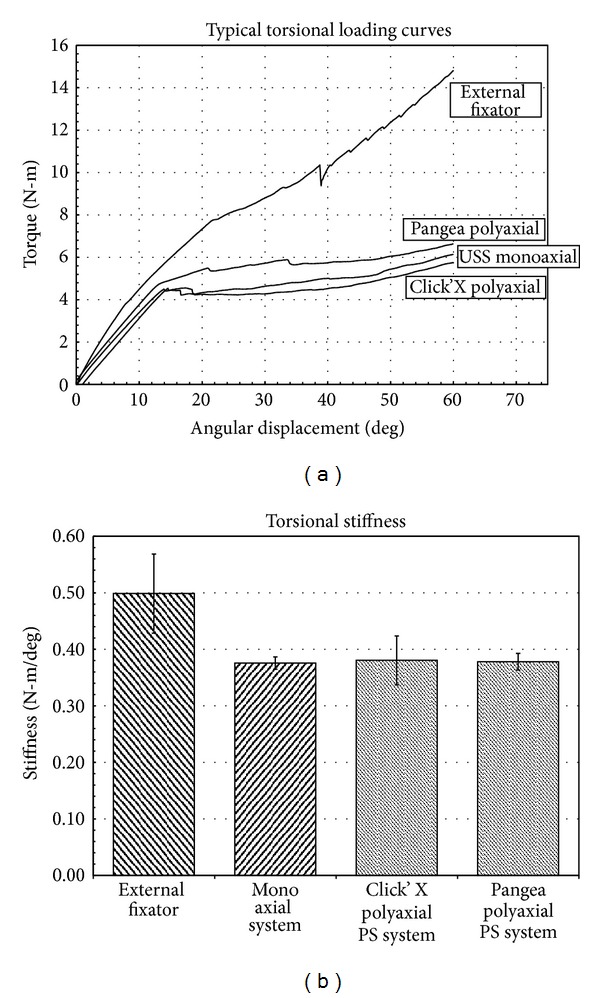
Typical torsion loading curves which show that the external fixator is stiffer than the pedicle screw systems.

The external fixator was significantly stiffer in torsion in the elastic range (0.5 ± 0.071 N-m/deg) than the monoaxial (0.376 ± 0.011 N-m/deg), Click-x (0.382 ± 0.045 N-m/deg) or Pangea poly (0.378 ± 0.015 N-m/deg) systems (*P* < .05) (Figure 4). Yield torques were similar for all constructs (Figure 5). The weak points in the constructs were the long Schanz pins that bent in the external fixator and the 6 mm rod and the screw-rod interface. The external fixator bent through the long schanz pins and the pedicle screw constructs moved through the 6 mm bar or the screw rod connector interface.

## 4. Discussion

Historically pelvic fractures have been treated by a variety of closed methods including slings and skeletal traction. With the advent of external fixation for the management of these injuries, improved functional outcomes have been achieved. The application of an external frame anteriorly restores stability in partially stable pelvic fractures [14]. Unstable fractures require anterior and posterior fixation [14, 15]. External fixation plays a role in the acute management of pelvic fractures. However, its role in the definitive management of these injuries is limited [14]. When used for definitive management, the fixator is retained for eight to twelve weeks which allows sufficient healing to occur. Prolonged use of pelvic external fixation leads to higher incidence of complications. There are reports of a marked difference in the complication rate between temporary and definitive fixators (21% and 62%, resp.) [16].

Using the principles of external fixation, a construct was designed to be placed under the skin which minimizes the possibility of pin tract infection and loosening [1]. This frame (infix) allows patients to sit upright, turn over from side to side, and lie in the prone position [8]. Pedicle screw systems have been reported to be useful for posterior pelvic fixation in scoliosis surgery and in posterior fractures [17], but the ability to place constructs anteriorly required anatomic dissections, examining patients, a definition of the “Bikini Area” as an anatomically stable area in sitting and standing and ultrasound confirmation once placed in patients [6]. It also required the knowledge that pedicle screw fixators were comparable to the standard of care, the external fixator [11, 12]. Results from the present study show that the pedicle screw systems had significantly higher stiffness than the external fixator to counteract distraction, which is the main function of anterior external pelvic fixation. The monoaxial pedicle screw system proved to be superior to the polyaxial screw systems, when tested up to 75 mm of distraction. The use of monoaxial screws to the uninitiated surgeon who tries to use this construct may be challenging since precise placement of the screws is mandatory for positioning of the connecting rod. Conversely, polyaxial screws allow some room for inaccuracies in their placement. It was found that the polyaxial screw constructs tested were still stiffer than the external fixator in distraction up to 40 mm of displacement or 157 ± 4 N at 45 mm for Click-x and 138 ± 6 N at 40 mm for Pangea after which the rod slid in the screw head. It is rare that the constructs would experience this amount of displacement; however, it has been reported [13] and may be one of the reason that in APC3 injuries, some constructs have demonstrated slippage or failure [8]. We have used monoaxial screws in situations that require excessive tension or added C-rings (Synthes Spine) on the outside of the polyaxial screws where one might see excessive tension or on the inside for lateral compression injuries. This was performed in 2 cases where the construct failed, but the original belief was that the failure was due to cross threading the caps in excessively large individuals [8]. Owen et al. [13] have suggested using 2 infix devices for this type of problem. In these cases, we recommend using monoaxial screws or reinforcing c-rings with polyaxial screws, which are both cheaper than 2 infix devices. Perhaps new implants should be designed to counteract the forces seen in extreme cases and the standard polyaxial pedicle screw implants may be suboptimal in some APC3 injuries.

When tested in torsion, the external fixator showed significantly higher peak torque and torsional stiffness compared to both the monoaxial and polyaxial screw systems. Yield loads for torsion however were similar for all four constructs. Clinically, the vertical displacement of pelvic fractures is unable to be controlled with anterior external fixation alone and posterior fixation is what surgeons use to hold the pelvis in reduction [14].

We have used infix acutely in several damage control situations once we became comfortable with the technique. Monoaxial screws were generally used in these instances as the construct appeared to be superior to the 2 pin external fixator. The construct remained in place until the definitive procedure when we loosened the infix, used the screws to help reduce the posterior injury, fixed the posterior pelvis, and readjusted the infix. In order to consider this, one has to have a set readily available and be very familiar with the technique, otherwise, it is best to use equipment that the surgeon is familiar with.

This study was performed with ultra-high molecular weight polyethylene blocks and simulations of the motions that the constructs would have to resist and is thus limited. The goal was to compare the two pin external fixator verses the pedicle screw fixator construct and not so much the bone screw interface, which appears to have been eliminated by using polyethylene. The screws used for the internal fixation construct, USS monoaxial, Click-x polyaxial, and Pangea polyaxial have thread designs that are made to increase purchase in cancellous bone such as a vertebral body or pelvis. This may also increase the strength of these devices verses the standard Schanz pin thread for the external fixator.

## 5. Conclusion

The biomechanical stability of this novel supra-acetabular internal fixation technique when constructed with traditionally available spinal instrumentation is comparable in stiffness to a two pin external fixator in distraction but less so in torsion. In addition, polyaxial constructs behaved favorably in stiffness testing when compared to monoaxial constructs and the external fixator in distraction. Both of these internal fixation constructs were less stiff than the external fixator in torsional testing. Testing to failure found that polyaxial screws may slip after 4 cm of distraction when the monoaxial screws and external fixator do not. This may be clinically relevant in extreme cases, and the use of monoaxial pedicle screws or to reinforce the polyaxial construct with C-rings on either side is recommended to prevent this. The development of a specific implant for anterior subcutaneous internal fixation may prove beneficial especially one that is able to counteract torsional forces.
